# A Plant-Derived Morphinan as a Novel Lead Compound Active against Malaria Liver Stages 

**DOI:** 10.1371/journal.pmed.0030513

**Published:** 2006-12-26

**Authors:** Maëlle Carraz, Akino Jossang, Jean-François Franetich, Anthony Siau, Liliane Ciceron, Laurent Hannoun, Robert Sauerwein, François Frappier, Philippe Rasoanaivo, Georges Snounou, Dominique Mazier

**Affiliations:** 1 Laboratoire de Chimie et Biochimie des Substances Naturelles, Muséum National d'Histoire Naturelle, Paris, France; 2 Inserm U511, Université Pierre et Marie Curie-Paris, Centre Hospitalier Universitaire Pitié-Salpêtrière, Paris, France; 3 Université Pierre et Marie Curie-Paris, Faculté de Médecine Pitié-Salpêtrière, Paris, France; 4 AP HP, Service de Chirurgie Digestive, Hépato-Bilio-Pancréatique et Transplantation Hépatique, Centre Hospitalo-Universitaire Pitié-Salpêtrière, Paris, France; 5 University Medical Center St. Radboud, Department of Medical Microbiology, Nijmegen, The Netherlands; 6 Laboratoire de Pharmacognosie Appliquée aux Maladies Infectieuses, Institut Malgache de Recherches Appliquées, Antananarivo, Madagascar; 7 Laboratoire de Parasitologie Comparée et Modèles Expérimentaux, Muséum National d'Histoire Naturelle, Paris, France; 8 Assistance Publique-Hôpitaux de Paris, Centre Hospitalo-Universitaire Pitié-Salpêtrière, Paris, France; University of London, United Kingdom

## Abstract

**Background:**

The global spread of multidrug–resistant malaria parasites has led to an urgent need for new chemotherapeutic agents. Drug discovery is primarily directed to the asexual blood stages, and few drugs that are effective against the obligatory liver stages, from which the pathogenic blood infection is initiated, have become available since primaquine was deployed in the 1950s.

**Methods and Findings:**

Using bioassay-guided fractionation based on the parasite's hepatic stage, we have isolated a novel morphinan alkaloid, tazopsine, from a plant traditionally used against malaria in Madagascar. This compound and readily obtained semisynthetic derivatives were tested for inhibitory activity against liver stage development in vitro *(P. falciparum* and *P. yoelii)* and in vivo *(P. yoelii)*. Tazopsine fully inhibited the development of P. yoelii (50% inhibitory concentration [IC_50_] 3.1 μM, therapeutic index [TI] 14) and P. falciparum (IC_50_ 4.2 μM, TI 7) hepatic parasites in cultured primary hepatocytes, with inhibition being most pronounced during the early developmental stages. One derivative, *N*-cyclopentyl-tazopsine (NCP-tazopsine), with similar inhibitory activity was selected for its lower toxicity (IC_50_ 3.3 μM, TI 46, and IC_50_ 42.4 μM, TI 60, on P. yoelii and P. falciparum hepatic stages in vitro, respectively). Oral administration of NCP-tazopsine completely protected mice from a sporozoite challenge. Unlike the parent molecule, the derivative was uniquely active against *Plasmodium* hepatic stages.

**Conclusions:**

A readily obtained semisynthetic derivative of a plant-derived compound, tazopsine, has been shown to be specifically active against the liver stage, but inactive against the blood forms of the malaria parasite. This unique specificity in an antimalarial drug severely restricts the pressure for the selection of drug resistance to a parasite stage limited both in numbers and duration, thus allowing researchers to envisage the incorporation of a true causal prophylactic in malaria control programs.

## Abstract 2

A derivative of a morphinan alkaloid, tazopsine, from a plant used against malaria in Madagascar, is active against the hepatic stages of*Plasmodium* species.

## Editors' Summary

**Background.:**

The parasite that causes malaria has quickly developed resistance to many of the drugs that are commonly used to treat this disease. As a result, new drugs and drug combinations are needed. In some parts of the world where antimalarial drugs are failing due to resistance, or are not available to everyone, people often turn to traditional herbal remedies instead. These traditional plant remedies can be a useful starting point for development of new drugs, but the process of developing effective new drugs from plant remedies is long and complicated. An important initial step is to isolate and identify the active compounds from plants and then see how well these compounds perform against malaria parasites in laboratory tests. If the tests are successful, such compounds could then progress to experiments in animals and possibly eventually human trials. One plant used widely in Madagascar for treatment of malaria is *Strychnopsis thouarsii;* the traditional remedy consists of the plant stem bark boiled in water.

**Why Was This Study Done?:**

The group of researchers doing this study wanted to discover candidates for new malaria drugs. They therefore wanted to find out which molecular compounds in the stem bark of S. thouarsii contained antimalarial activity, and what particular stage of the malaria parasite's life cycle these compounds had an effect on. The researchers suspected that the agents in this plant bark had some activity against the “liver stage” of malaria infection in humans. This is the first stage of infection, after a person has been bitten by a malaria-infected mosquito, and before blood cells are invaded by malaria parasites (which then causes the disease symptoms). Very few drugs currently in existence have an effect on the “liver stage” of infection, but activity at this stage would be tremendously useful because it could mean a drug is better for prevention of malaria than others in existence.

**What Did the Researchers Do and Find?:**

First, the researchers wanted to take the traditional herbal remedy—of S. thouarsii bark boiled in water—and find out precisely which molecule in that remedy was responsible for the antimalarial activity. They therefore used a method called chromatography to progressively separate the herbal extract into its distinct components. At each stage of separation, the extract was checked for activity against malaria using a laboratory test. Inactive extracts were disregarded, and the active component then taken on to a further separation round. After many rounds of separation and testing, the researchers got down to a single, apparently new, molecule that was active against malaria in the laboratory test, and this molecule was named tazopsine (in the Malagasy language the word Tazo refers to malaria). In order to find out how effective the molecule was at killing malaria parasites, the researchers took human or mouse liver cells cultured in the laboratory, infected them with malaria parasites (either the malaria parasite that normally infects humans, or a related species that infects mice), and then added tazopsine at different concentrations. The compound completely killed the malaria parasites even at very low concentrations, and had activity against malaria infecting either liver cells or red blood cells. Tazopsine was then given to mice injected with a species of the malaria parasite. The compound protected most mice against malaria infection when it was used at a dosage level lower than the toxic dose. The researchers then tried making a series of different variants of tazopsine in the hope that some variants would be less toxic, but equally active as, the original compound. They found one variant, named NCP-tazopsine, that was much less toxic but just as active as tazopsine, but only against the malaria infecting liver cells.

**What Do These Findings Mean?:**

In these experiments a new molecule, tazopsine, was discovered from a Malagasy plant, and it was found to be active against liver-stage malaria parasites, in laboratory experiments and in mice. This molecule or variants of it could in future become candidate antimalarial drugs in humans. However, much work would need to be done before testing could get to that stage. Different variants of molecules related to tazopsine would need to be tested to find one that has low toxicity, and these variants would need to be fully evaluated in animals to see how they are handled in the body before any trials could begin in humans.

**Additional Information.:**

Please access these Web sites via the online version of this summary at http://dx.doi.org/10.1371/journal.pmed.0030513

- The World Health Organization publishes a minisite containing links to information about all aspects of malaria worldwide, including treatment, prevention, and current programmes for malaria control
- Medicines for Malaria Venture is a collaboration between public and private organizations (including the pharmaceutical industry) that aims to fund and manage the development of new drugs for treatment and prevention of malaria
- Wikipedia entries for drug discovery and drug development (note: Wikipedia is an internet encyclopedia that anyone can edit)

## Introduction

Malaria re-emerged abruptly in the highlands of Madagascar in the mid-1980s and was associated with large-scale mortality and morbidity in the nonimmune inhabitants [1]. Shortage of antimalarial drugs, mainly chloroquine, led the population to rely on traditional herbal remedies. In a subsequent large-scale ethnobotanical survey, 229 plant species, of which 30% are endemic to Madagascar, were reported to possess antimalarial activity [2], although the manner in which they are administered was not recorded. One of the endemic plants, Strychnopsis thouarsii (Menispermaceae) [3,4], was selected for further investigations, because it is the sole ingredient of a widely used remedy reputed to provide protection specifically against malaria [5].

The traditional decoction, prepared simply by boiling S. thouarsii stem bark in water, showed only weak activity in vitro against the FcB1 strain of P. falciparum erythrocytic stages (50% inhibitory concentration [IC_50_] 34.0 ± 9.4 μg/ml). Given that some medicinal preparations are also used as a preventive (for example, during epidemics), we hypothesized that this particular remedy, and others that are similarly poorly active against the blood stage parasites, might actually be active against the initial pre-erythrocytic (PE) phase of the infection [6]. This phase represents the invasion of hepatocytes by the sporozoites inoculated by the mosquito and the full development of the hepatic parasites from which the pathogenic erythrocytic phase originates. Full inhibition of the PE stages of the infection provides true causal prophylaxis whereby the blood stage infection and its associated clinical manifestations would be totally prevented. For P. vivax and *P. ovale,* in which dormant hepatic forms, the hypnozoites, can give rise to relapses in humans, drugs active against hypnozoites would provide a radical cure of the infection. In drug discovery programs, routine screening for anti-PE stage activity is seldom carried out, partly because these stages are clinically silent, but mainly because access to PE parasites is costly and restricted to a few laboratories. We aimed to establish whether the traditional remedy prepared from S. thouarsii contained agents active against *Plasmodium* hepatic stages, and if so, to isolate them and explore their suitability as future lead compounds.

## Methods

### Drugs

Tazopsine, [α]_D_^22^ −46° (*c* 0.5, MeOH) and HRCIMS *m/z* 350.1608 [M+H]^+^, was purified from Strychnopsis thouarsii stem bark collected in the eastern rain forest of Madagascar at Andasibe National Park. *N*-cyclopentyl-tazopsine (NCP-tazopsine), [α]_D_^22^ −30° (*c* 0.03, MeOH) and HRCIMS *m/z* 418.2239 [M+H]^+^, was obtained from tazopsine by a classical method of amine reductive alkylation [7]. Briefly, 200 μl (2.3 mmol) of cyclopentanone and 150 mg (2.4 mmol) of sodium cyanoborohydride (Fluka, Buchs, Switzerland) were added successively to 700 mg (2 mmol) of tazopsine solubilized in 5 ml of absolute methanol and stirred at room temperature under argon for 24 h. After removal of the solvent under reduced pressure, the residue was acidified with 1 N HCl, then basified with NH_4_OH, dried and purified by a silica gel column chromatography eluted with dichloromethane–methanol–20% ammonia (90-10-1). A total of 675 mg (1.62 mmol) of NCP-tazopsine were obtained (81% yield). Primaquine was purchased from Sigma (Saint-Quentin Fallavier, France).

### In Vitro Assay for P. yoelii and P. falciparum Liver Stage Inhibition

P. yoelii (265 BY strain) and P. falciparum (NF 54 strain) sporozoites were obtained by dissection of infected Anopheles stephensi salivary glands. Primary mouse hepatocytes were isolated as previously described [8] and seeded in eight-well Lab-Tek plastic chamber slides (VWR, Fontenay-sous-Bois, France) previously coated with rat-tail collagen I (BD Biosciences, Le Pont de Claix, France) at a density of 10^5^ cells per well. Mouse hepatocytes were cultured at 37 °C in 5% CO_2_ in William's E medium supplemented with 10% fetal calf serum, 1% L-glutamine, 1% sodium pyruvate, 1% insulin-transferrin-selenium, 1% nonessential amino acids, and 1% penicillin-streptomycin (all from Invitrogen, Cergy-Pontoise, France), for 24 h before inoculation of P. yoelii sporozoites (10^5^ per well). Human liver fragments were collected following informed consent provided from patients undergoing a partial hepatectomy. The collection and use of these tissues were undertaken in accordance with French Government ethical regulations. Primary human hepatocytes were isolated from the healthy parts of these liver biopsies as previously described [9]. Human hepatocytes were seeded in LabTek plastic chamber slides coated with collagen, at a density of 1.8 × 10^5^ cells per well and cultured at 37 °C in 5% CO_2_ in the same medium as above, supplemented with 10^−7^ M dexamethasone (Sigma) and 2% DMSO after complete cell adherence (12–24 h), for at least 48 h before inoculation of P. falciparum sporozoites (10^5^ per well). For a standard assay, the plant extract, the purified compound, or primaquine was solubilized in DMSO, further diluted in culture medium (equal DMSO concentrations of <0.3% per well), and then added to hepatocyte cultures at the time of sporozoite inoculation. Three hours later, after sporozoite penetration into hepatocytes, cultures were washed and further incubated in the presence of the drug. Culture medium containing the appropriate drug concentration was changed daily until 48 h for *P. yoelii,* or until 5 d for P. falciparum. Parasites were quantified on the last day of incubation. For the in vitro timing experiments, tazopsine treatment of P. yoelii and P. falciparum cultures was undertaken for different periods during the cultivation: 0 h to 3 h, 3 h to 24 h, or 24 h to 48 h after P. yoelii sporozoite inoculation (quantification at 48 h), or 0 h to 2 d, or 3 d to 5 d after P. falciparum sporozoite inoculation (quantification at 5 d). Parasite quantification was done by immunofluorescence analysis following fixation of the cultures with cold methanol and parasite-specific staining by a mouse polyclonal serum raised against the P. falciparum heat shock protein 70 that also cross-reacts with the homologous protein of *P. yoelii,* and revealed with FITC-conjugated goat anti-mouse immunoglobulin (Sigma) [8]. Cells were stained with Evans Blue (0.4%), and parasites and cells nuclei were stained with 1 μg/ml of diamidino-phenylindole (DAPI; Sigma). Parasite numbers were counted under a fluorescence microscope with a 25× light microscope objective. IC_50_ values, the drug concentration at which a 50% reduction in the number of parasites was observed, as compared to the number in the DMSO control cultures, were derived from two independent experiments in which each concentration was tested in triplicate wells.

### In Vitro Assay for Cell Viability

Primary hepatocytes of mice or humans were prepared as above and seeded in 96-well plates coated with collagen, at a density of 5 × 10^4^ and 7 × 10^4^ cells per well, respectively. Drugs were added to the culture medium, which was changed daily after 24 h or 48 h of incubation for mouse and human hepatocytes, respectively. After 48 h of drug exposure (mouse hepatocytes) or 5 d (human hepatocytes), 50 μl of neutral red (Sigma) at 0.02% in phosphate-buffered saline (PBS) were added per well and incubated for 24 additional hours [10]. Cultures were then washed with PBS and the neutral red extracted with 1% SDS from viable cells and quantified by measuring the OD_540_ on a microplate autoreader photometer (BioTek Instruments, Winooski, Vermont, United States). The 50% toxic concentration (TC_50_) values, the drug concentration at which a 50% reduction of red neutral incorporation was observed, as compared to that incorporated in the DMSO control cultures, were derived from two independent experiments in which each concentration was tested in quadruplicate.

### Statistical Methods for IC_50_ and TC_50_ Estimations

IC_50_ and TC_50_ were calculated by nonlinear regression using four-parameter logistic curves on the SigmaPlot 10.0 software (http://www.systat.com). Each IC_50_ or TC_50_ is expressed as a mean between two independent experiments, followed by the standard error of the mean.

### In Vitro Assay for P. falciparum Blood Stages Inhibition

P. falciparum clone 3D7 (chloroquine sensitive) and clone FCR3 (chloroquine resistant) erythrocytic stages were cultured as described [11], and drug antiplasmodial activity was assessed by the standard method [12] with minor modifications. Briefly, the compounds tazopsine and NCP-tazopsine solubilized in DMSO were further diluted in RPMI before adding to parasite cultures (0.5% parasitemia, mainly young trophozoites, and 1.8% final hematocrit), in a 2-fold dilution series. Plates were maintained for 24 h at 37 °C in 5% CO_2_. Then 0.5 μCi of [^3^H]-hypoxanthine was added to each well, and cultures were incubated for a further 24 h. The growth inhibition was determined by comparison of the radioactivity incorporated into the treated cultures with that into the control cultures, by liquid scintillation counting.

### In Vivo Assay for Activity on P. yoelii Infections

All animal were kept and used in accordance with institutional guidelines and European regulations. Female 6-wk-old Swiss mice (René Janvier, Le Genest-Saint-Isle, France) weighing 24–31 g were randomly allotted to five groups. The drug to be tested was administered orally. Tazopsine was diluted in sterile water and NCP-tazopsine in sterile water with 10% of Tween 80 (Aldrich, Le Chesnay, France). The drugs were administrated on days −1, 0, +1, +2 (+40 h), and the mice challenged on day 0 by retro-orbital injection of P. yoelii (265 BY strain) sporozoites. Group 1 received 100 mg/kg/day of tazopsine (*n* = 5), and group 2 received sterile water at the same times (controls, *n* = 5); groups 1 and 2 were challenged with 4,000 sporozoites. Group 3 received 100 mg/kg/d of NCP-tazopsine (*n* = 5), group 4 received 200 mg/kg/d of NCP-tazopsine (*n* = 8), and group 5 received 10% Tween in sterile water (controls, *n* = 5); groups 3, 4, and 5 were challenged with 10,000 sporozoites. Parasites were enumerated by blinded microscopic examination of Giemsa-stained tail-blood smears collected from day 2 to day 23 postinoculation. A mouse was considered negative when no parasites could be observed in 20,000 red blood cells (50 fields under a 50× light microscope objective) on any day during the period of observation. The results presented were derived from two independent duplicate experiments.

In order to test for inhibitory activity against P. yoelii blood stages, four groups of five naive mice were inoculated (intraperitoneally) with 10^6^ *P. yoelii–*infected red blood cells on day 0. Three hours later, one group was treated with tazopsine at 100 mg/kg, another with NCP-tazopsine at 200 mg/kg, whereas the other two groups acted as controls (respectively, water and 10% Tween 80). The doses were repeated daily until day +3. For the group treated with NCP- tazopsine and its control group, the animals were anesthetised 2 h following the administration of the last dose (day +3), and placed for 30 min over a cage with 100 female laboratory-bred A. stephensi. Oocysts were enumerated in 30 mosquito midguts dissected 8 d later, and the number of sporozoites in the salivary glands dissected from 30 insects was ascertained a further 7 d later. All the experiments were performed in two independent duplicates, and enumeration of parasites in blood smears and mosquitoes was conducted in a blinded fashion.

### Real-Time PCR Quantification of P. yoelii Parasites in Mouse Livers

Female 6-wk-old Swiss mice weighing 24–31 g were randomly allotted to three groups. The drug to be tested was administrated orally on days −1, 0, and +1. Group 1 received 100 mg/kg/d of NCP-tazopsine (*n* = 4), group 2 received 200 mg/kg/d of NCP-tazopsine (*n* = 6), whereas the control group received Tween 80 at 10% in sterile water (*n* = 5). Mice were challenged on day 0 by retro-orbital injection with 120,000 P. yoelii sporozoites and sacrificed 42-h postinoculation. A piece of liver (0.2 g) was harvested and total RNA extracted using the Micro to Midi kit (Invitrogen) according to the manufacturer's instructions. The detection and quantification of liver stage parasites were done as described [13] with minor modifications. Five micrograms of total RNA were treated with DNase from the Turbo DNA-free kit (Ambion, Huntingdon, United Kingdom) and reverse transcribed by Superscript II (Invitrogen) according to the manufacturer's instructions. A total of 6 μl of the 300 μl obtained (an equivalent to 100 ng of the starting total RNA) were used per TaqMan PCR assay (MX4000 multiplex quantitative PCR system; Stratagene, La Jolla, California, United States). Gene-specific P. yoelii primers (forward 5′-TTAGATTTTCTGGAGACAAACAACT, reverse 5′-TCCCTTAACTTTCGTTCTTGAT; Invitrogen) and a probe (5′-6FAM-CGAAAGCATTTGCCTAAAATACTTCCAT-BHQ1; MWG Biotech, Ebersberg, Germany) were designed from the P. yoelii 265BY 18S rRNA sequence using the Primer Express software (PE Applied Biosystems, Courtaboeuf, France). Lack of nonspecific amplification with mouse genomic DNA was determined in preliminary experiment (unpublished data). Mouse β-actin primers (forward 5′-ACGGCCAGGTCATCACTATTG, reverse 5′-CAAGAAGGAAGGCTGGAAAAG; Invitrogen) and a probe (5′-HEX-CAACGAGCGGTTCCGATGCCC-BHQ2; MWG Biotech) were designed and used for normalization. Purified plasmids (pCRII-TOPO) containing P. yoelii 18S rRNA and mouse β-actin PCR fragments cloned with the TOPO TA cloning kit (Invitrogen) were used in a 10-fold dilution series to obtain a standard curve. All TaqMan test samples and plasmid standards were performed in triplicate. Absolute transcript copy number for each gene was calculated based on their external standard curves. For each sample, P. yoelii rRNA 18S absolute copies were normalized to 10^6^ copies of mouse β-actin.

### Statistical Analysis

Statistical analysis was undertaken by one-way analysis of variance (ANOVA) tests coupled to Tukey HSD tests; in this latter test, the exact *p-*value is given only if it exceeds 0.01.

## Results

**Figure 1:**
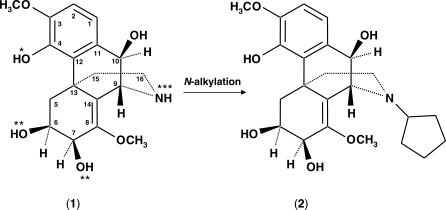
Structure of Tazopsine Tazopine is shown on the left. The chemically modified substituents are indicated by asterisks (left): a single asterisk (*) indicates 4-OH protected by 4-*O*-alkylations; double asterisks (**) indicate 6,7-diol protected by an acetonide ketal; and triple asterisks (***) indicate secondary amine substituted by *N*-acetylation and *N*-alkylations. Structure of the semisynthetic alkaloid, NCP-tazopsine is shown on the right.

The activity of the S. thouarsii decoction was assessed in a standard bioassay of cultured mouse primary hepatocytes infected with P. yoelii sporozoites [14,15]. Parasite inhibition was observed in the exposed cultures (IC_50_ 8.5 ± 0.7 μg/ml), with hepatic forms completely eliminated at concentrations of 20 μg/ml or greater. Toxicity, as assessed by red neutral incorporation into the uninfected primary hepatocytes, was absent at the highest concentrations tested (100 μg/ml). In order to identify the compound(s) responsible for this activity, we conducted a bioassay-guided fractionation (Figure S1). The activity resided in the methanolic, but not the ethanolic extract (consistent with a highly polar compound), with an IC_50_ of 12.5 ± 3.5 μg/ml. Successive chromatographic separations, monitored by the in vitro P. yoelii liver stage inhibition assays, led to the isolation of a pure active compound that we named tazopsine which represented the major constituent of the dry material obtained from the traditional decoction (10.3% w/w) (Figure S2). The structure of tazopsine was solved by two-dimensional ^1^H and ^13^C nuclear magnetic resonance (NMR) spectroscopy (Table S1). It consists of a morphinan skeleton bearing two methoxyl groups at positions 3 and 8, and four hydroxyl groups at positions 4, 6, 7, and 10 (Figure 1), with a molecular weight of 349. To the best of our knowledge, this structure had not been previously described.

### Antimalarial Activity of Tazopsine

**Figure 2:**
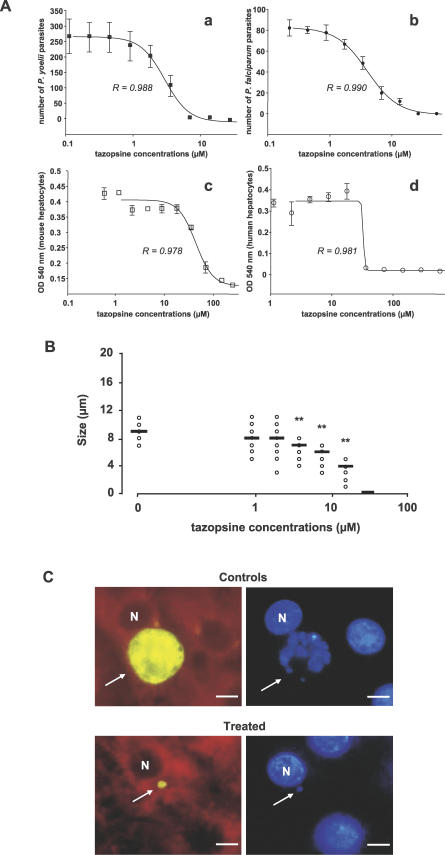
Inhibitory Activity of Tazopsine on *Plasmodium* Hepatic Stages in vitro (A) Representative curve of the dose-dependent inhibition of tazopsine (the number of parasite forms in test wells divided by the number of parasites in control wells) for graph a, P. yoelii (filled squares [], average number in control wells = 274), and graph b, P. falciparum (filled circles [•], average number in control wells = 83). Toxicity as assessed by the neutral red assay is presented for graph c, mouse (open squares [□]), or graph d, human (open circles [○]) primary hepatocytes. Results are means ± standard deviation within triplicates of one experiment. *R* represents the factor of regression calculated by SigmaPlot. (B) Dose-dependent influence on the size of surviving P. falciparum hepatic forms, (21 forms were counted for each point, and the bar represents the median value). Significance was calculated in one-way ANOVA analysis for the whole dataset (*p* < 0.0001), coupled to a Tukey HSD test: double asterisks (**) indicate *p* < 0.01. (C) P. falciparum hepatic forms (arrow) from cultures treated with tazopsine (15 μM) for 5 d, or untreated control cultures. DAPI staining is shown in the right images, and P. falciparum–specific staining (anti-PfHSP70 antibodies) in the left images. N, hepatocyte nucleus. The bar represents 5 μm.

Purified tazopsine was found to be active against the liver stages of P. yoelii and those of P. falciparum. Total inhibition of cultured P. yoelii hepatic stages was obtained at 7.1 μM or greater (IC_50_ 3.1 ± 0.1 μM) in cultured primary hepatocytes (Figure 2A), yielding a therapeutic index (TI) of 14 (TC_50_ 43.7 ± 1.6 μM). For P. falciparum–infected primary human hepatocyte cultures [9], parasite development was fully inhibited at higher concentrations of 28 μM or greater (IC_50_ 4.2 ± 0.3 μM), and the corresponding TI using human primary hepatocytes (TC_50_ 28.4 ± 4.3 μM) was 7 (Figure 2A). Tazopsine was also active against the erythrocytic stages of P. falciparum in vitro (IC_50_ 4.7 ± 0.9 μM using the 3D7 line, IC_50_ 5.7 ± 2.1 μM using the FCR3 line).

**Figure 3:**
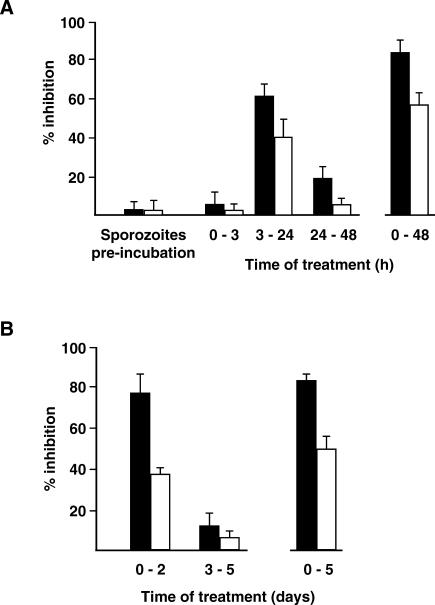
Susceptibility of *Plasmodium* Hepatic Stages to Tazopsine during Growth It should be noted that the in vivo duration of the hepatic phase of P. yoelii is 44 to 48 h, whereas that of P. falciparum is just under 6 d. (A) P. yoelii cultures were subjected to 3 μM (IC_50_; open bars) or 5 μM (IC_80_; filled bars) of tazopsine for different periods of the development. Preincubation of 80,000 sporozoites (Spz) in the presence of 5 μM or 100 μM of tazopsine for 1 h or 3 h, respectively, were carried out at room temperature prior to washing and being added to the mouse primary hepatocyte well. (B) P. falciparum cultures were subjected to 4 μM (IC_50_; open bars) or 10 μM (IC_80_; filled bars) of tazopsine for different periods of development. Inhibition was measured as the number of parasite forms in test wells divided by the number of parasites in control wells, for *P. yoelii,* the average number in control wells equaled 408, and for *P. falciparum,* 105. Results are means ± standard deviation.

Examination of cultures subjected to suboptimal inhibitory concentrations revealed that tazopsine inhibited growth because the surviving hepatic parasites were significantly smaller than those observed in control cultures (Figure 2B and 2C). In order to determine the point at which the liver parasites were most susceptible to inhibition by tazopsine, we exposed P. yoelii hepatic cultures to suboptimal inhibitory concentrations at the different phases of the infection: the first 3 h postinfection (PI), by which time the sporozoites have invaded the hepatocytes; from 3 to 24 h PI, at the end of which, nuclear division has just been initiated in the growing trophozoites; and finally, from 24 to 48 h PI when the schizonts were maturing to large multinucleate forms. Sporozoite invasion and differentiation into early hepatic stages were not affected by tazopsine, nor did pretreatment of sporozoites with tazopsine alter hepatocyte invasion efficiency (Figure 3A). Inhibition was the most pronounced during the early developmental stages of the hepatic parasite (3 to 24 h), with only residual inhibitory activity on growing young schizonts (24 to 48 h) (Figure 3A). In parallel experiments, P. falciparum hepatic stages were similarly found to be most susceptible to inhibition during the early stage of trophozoite development (Figure 3B). For both parasite species, the inhibitory effect was found to be dose dependent (Figure 3). It should be pointed out that hepatic stages of P. yoelii and P. falciparum differ in the time required for maturation into forms containing invasive merozoites. Development is completed at the earliest in 45 h for *P. yoelii,* and in 5.5 to 6 d for P. falciparum. For both species, maturation takes longer in cultured primary hepatocytes. Nonetheless, the P. yoelii model has been extensively used for biological, immunological, and chemotherapeutical investigations; and to a large extent, similar findings were observed when the corresponding studies were carried out using P. falciparum [16–18].

### Hepatic Stage Specificity and Protective Efficacy of NCP-Tazopsine

**Figure 4:**
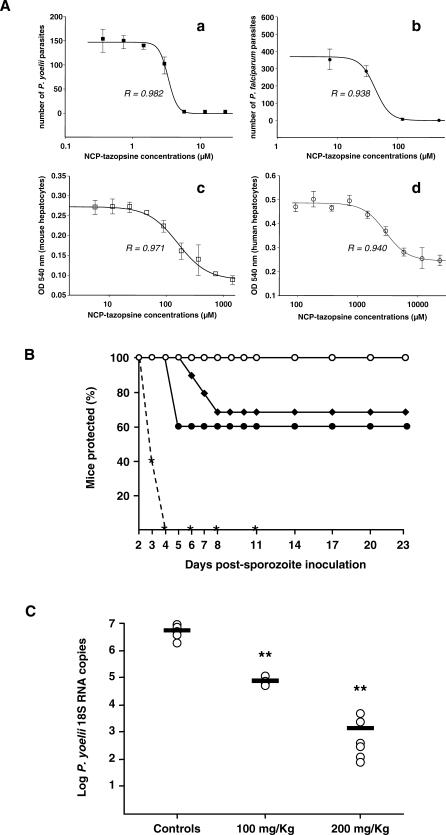
Inhibitory Activity of NCP-Tazopsine on *Plasmodium* Hepatic Stages in vitro and Protective Activity of Tazopsine and NCP-Tazopsine for P. yoelii Infections in vivo (A) Representative curve of the dose-dependent inhibition of NCP-tazopsine (the number of parasite forms in test wells divided by the number of parasites in control wells) for (graph a) P. yoelii (filled squares [], average number in control wells = 152), and (graph b) P. falciparum (filled circles [•], average number in control wells = 384). Toxicity is presented for (graph c) mouse (open squares [□]) or (graph d) human (open circle [○]) primary hepatocytes. Results are means ± standard deviation within triplicates of one experiment. (B) Proportion of mice that failed to acquire a blood-stage infection following sporozoite challenge through treatment with tazopsine (filled diamonds [♦], 100 mg/kg), NCP-tazopsine (filled circles [•], 100 mg/kg, and open circles [○], 200 mg/kg), distilled water or 10% Tween in distilled water (asterisks [*] indicate control groups). (C) Dose-dependent reduction of hepatic parasite load by NCP-tazopsine following sporozoite challenge. Bar represents the average value. Significance was calculated in one-way ANOVA analysis for the whole dataset (*p* = 0.0012), coupled to a Tukey HSD test: double asterisks (**) indicate *p* < 0.01.

Preliminary experiments established that tazopsine was toxic to mice at oral doses exceeding 100 mg/kg given daily over 4 d (the standard number of doses in this malaria model). Upon challenge with 4,000 P. yoelii sporozoites, protection from infection was obtained in 70% of the mice treated at 100 mg/kg daily for 4 d, whereas the remaining 30% developed a blood stage parasitemia, albeit their prepatent period (the time from inoculation until parasites become microscopically detectable) was extended by about 4 d over that of the controls (Figure 4). In order to determine whether protection from infection might be in part due to an additional effect of tazopsine on the blood stage parasite, groups of mice were treated as above and challenged on day 0 with P. yoelii–infected blood. No significant inhibition of the erythrocytic multiplication cycle could be detected (unpublished data). This confirmed that the protection was due to an inhibition of the hepatic stages.

The toxicity of tazopsine in mice and on cultured human cells was of concern. We, therefore, synthesized a series of derivatives from tazopsine, and those still found active against cultured P. yoelii (IC_50_ < 50 μM), were tested for toxicity. Of the seven substituents of tazopsine, four were targeted because they were amenable to functionalization by classical alkylation: the three hydroxyl groups at positions 4, 6, and 7, and the secondary amine (Figure 1). Whereas alkylation of the three hydroxyl groups led to a complete loss of activity, inhibition of liver stages was retained by seven of the ten semisynthesized *N*-alkylated derivatives. Their IC_50_ values, determined using the hepatic P. yoelii inhibition assay, ranged from 3.3 μM to 24 μM. NCP-tazopsine had the lowest IC_50_ (3.3 ± 0.05 μM) and the lowest toxicity to cultured primary mouse hepatocytes (TC_50_ 150.5 ± 9.5 μM). These values are a significant improvement on tazopsine, yielding a TI of 46 for NCP-tazopsine (Figure 4A). Dose-dependent inhibition of P. falciparum hepatic stages was also obtained with NCP-tazopsine, with an IC_50_ of 42.4 μM and a TC_50_ for primary human hepatocytes of 2,549.6 ± 307.6 μM, resulting in a TI of 60 (Figure 4A), again a more adequate value than that of tazopsine. Furthermore, NCP-tazopsine did not have any detectable effect on the multiplication in vitro–cultured erythrocytic *P. falciparum* (both the 3D7 and the FCR3 lines) up to concentrations of 600 μM. By way of comparison, the activity of primaquine, the only drug licensed for use against the parasite's hepatic forms, has been determined in parallel in the same model systems (Table 1). The IC_50_ for primaquine is close to 1 μM for both parasite species, and although it is significantly more active than NCP-tazopsine on *P. falciparum,* similar therapeutic indices were observed with the two molecules for this parasite species.

**Table 1:**
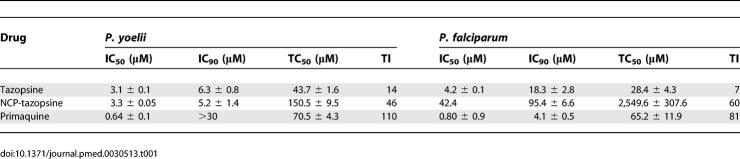
Comparative Inhibitory Activities of Tazopsine, NCP-Tazopsine, and Primaquine on Hepatic Stages Cultured in Primary Hepatocytes

Oral administration of NCP-tazopsine up to doses of 400 mg/kg for 4 d was not found to be toxic to mice. When mice treated with daily 200 mg/kg doses over 4 d were challenged with 10,000 P. yoelii sporozoites, none of the mice developed a blood stage infection (Figure 4B). At subdoses of 100 mg/kg for 4 d, 60% of the mice challenged were protected from infection, and the prepatent period of the nonprotected mice was extended by 2 d over controls, indicating a substantial reduction in the number of liver parasites [19]. Administration of the drug NCP-tazopsine at this fully protective dose to animals inoculated with P. yoelii–infected blood had no effect on blood stage parasite multiplication (unpublished data). Analysis of female A. stephensi mosquitoes fed on treated and untreated mice, revealed that NCP-tazopsine had no effect on the transmissibility of the parasite (unpublished data). These observations confirmed that NCP-tazopsine is only active against the hepatic phase of the life cycle. This result was further confirmed through quantification by real-time PCR, performed on RNA purified from the livers of another group of mice challenged with a massive sporozoite dose (120,000 parasites) and treated with NCP-tazopsine. As compared to controls, parasite loads at 42 h PI in the animals treated with the fully protective dose were reduced by 99.98%, and those at the suboptimal dose by 98.58% (Figure 4C).

## Discussion

We have described a naturally occurring morphinan, tazopsine, that can be readily isolated in high yields (0.56% w/w) from the stem bark of S. thouarsii. This compound represents a novel class of antimalarial drugs and possesses outstanding inhibitory activity against the *Plasmodium* hepatic stages (Patents: Priority Europe No 04 291 055.4 April 22 2004; International Patent Application 21 April 2005 No PCT/EP 2005/005239). Tazopsine represents one of the first plant-derived molecules to be chemically characterized with activity against the parasite's hepatic stage.

Exposure to tazopsine prevented the in vitro development of P. falciparum and *P. yoelii,* although with relatively low therapeutic indices. The semisynthetic derivative NCP-tazopsine, prepared through simple amino alkylation, showed substantially improved therapeutic indices in vitro and fully protected mice against a large sporozoite challenge when administered orally. The IC_50_ values and the therapeutic indices of NCP-tazopsine do not differ substantially from those of the licensed primaquine (Table 1). It is noteworthy that the few chemical modifications of tazopsine attempted to date have led to a compound, NCP-tazopsine, with significantly reduced toxicity and which have lost activity against P. falciparum blood stages in vitro. It is likely that further derivatizations, to which tazopsine is readily amenable, would generate one or more compounds with improved pharmacological characteristics. Other morphinans, such as the opioids morphine, codeine, and thebaine, were not found to be inhibitory when tested against the P. yoelii liver stages (IC_50_ > 100 μM). Indeed these morphinans differed from tazopsine and NCP-tazopsine mainly by the inverse absolute configuration of asymmetric carbons on the piperidine ring. It would thus seem that the known targets of morphine-related compounds are not implicated in the mechanism of action of tazopsine and NCP-tazopsine, which remains at present speculative. Classical biochemical and pharmacological investigations of *Plasmodium* liver stages are severely hampered by the nonavailability of purified infected hepatocytes.

The urgent need to counter the emergence and global spread of drug-resistant P. falciparum parasites has favored the search for novel blood schizontocides to the detriment of causal prophylactic agents. Furthermore, given the logistic obstacles to screening compounds for activity against the hepatic stage, namely the simultaneous availability of primary hepatocytes and sporozoites, only a minor fraction of the compounds screened for anti–asexual-stage activity are assayed against the infected hepatocyte. Consequently, there is a limited number of drugs effective against the *Plasmodium* liver stage parasite. The deployment of primaquine, the only drug specifically developed to inhibit the liver infection, has been curtailed by the associated toxicity, poor compliance, and increased risk of hemolysis when administered to persons with glucose-6-phosphate dehydrogenase deficiency. This latter problem will also affect the two related synthetic 8-aminoquinolines, bulaquine [20] and tafenoquine [21], presently undergoing clinical trials. Antifolates and atovaquone, primarily used in combination to treat the blood stage infection, were also shown to be active against the infected hepatocyte. However, the high prevalence of resistant parasites to the former, and the ease with which resistance arises to the latter, limit the prophylactic usefulness of these drugs. Quinine, chloroquine, mefloquine, and artemisinin-based compounds, have little or no efficacy against the hepatic parasite. Experimental compounds belonging to other chemical classes, e.g., antibiotics [22], antihistaminics [23], or anticancer drugs [24] have been reported to have activity against the PE stages of *Plasmodium*. Most of these were tested only on rodent malaria parasites and in which they were also active against the blood stages. Two quinoline esters prevented relapse of P. cynomolgi in monkeys [25], as did two imidazolidinedione compounds prepared from the antifolate chlorproguanil [26]. Although the first had additional activity against blood stage parasites, the two imidazolidinedione molecules proved not to inhibit the erythrocytic stages of P. berghei in mice nor those of P. falciparum in vitro [27]. The hepatic stage specificity of the imidazolidinediones and NCP-tazopsine places these lead molecules from two distinct chemical classes into a novel category of antimalarial compounds with true causal prophylactic activity.

Although tazopsine constitutes a suitable lead candidate for further optimization, there are a number of issues that would need to be addressed before it reaches the status of a drug candidate. Toxicity is a very important parameter since a drug aimed for prophylactic use will by definition be administered over long periods of time. Two aspects of the results are of some encouragement in this context. Derivatives, such as NCP-tazopsine, that retain inhibitory activity yet have significantly reduced toxicity as compared to the parent compound tazopsine, were readily obtained. The recent development of high-throughput screening of drug activity on *Plasmodium* hepatic stages [28] should enhance lead optimization, and provide candidates for pharmaceutical optimization (adsorption, distribution, metabolism and excretion [ADME]). Full protection from an in vivo challenge was afforded by oral administration of NCP-tazopsine, although it remains to be demonstrated that a similar route of intake will be equally effective in primates. As compared to blood schizontocides in which inhibitory activity is observed in the low nM range, the μM concentrations of tazopsine and NCP-tazopsine required for the inhibitory activity of hepatic stages in vitro were relatively modest. The equivalent values for primaquine (0.64 μM and 0.8 μM for the P. yoelii and P. falciparum liver stages in vitro), were also close to 1 μM (Table 1). These high concentrations, relative to blood schizontocides, might reflect the fact that the target of the drug is found within the cells of the liver, a metabolically complex and active organ in part devoted to detoxification.

Historically, a primary aim of antimalarial drug research has been to develop a true causal prophylactic. These efforts were much diminished by the introduction of the remarkably successful chloroquine after the second World War, although the high rate of P. vivax relapses in military personnel returning to the United States from Korea in the early 1950s led to primaquine [29], a drug still primarily used to effect the radical cure of P. vivax and P. ovale by preventing relapses due to hypnozoites. The fact that all antimalarials used to date with true prophylactic activity also inhibit the erythrocytic stages precludes any consideration of mass administration. This is because exposure of blood stage parasites, in which cyclical multiplication leads to high parasite loads and the persistence of millions of parasites in the human host for many weeks or months, to subcurative drug concentrations exerts substantial pressure that would lead to the selection and dissemination of drug-resistant parasites. This exposure to selective drug pressure constitutes a major impediment to efforts aimed at developing novel causal prophylactic compounds. A drug active solely against the liver stages would exert only marginal selective pressure, since a single infective bite leads to an average of a few dozen hepatic stage parasites that usually persist for less than 2 wk, during which they undergo a single multiplicative expansion. The deployment of such a drug would be especially valuable in containing the spread of the disease in areas of emerging or epidemic malaria, and in residents subjected to seasonal transmission. It would be the ideal choice for transient visitors to endemic areas, such as displaced populations, tourists, and the military. In populations residing in areas of higher endemicity, the drug would help reduce the effective dose of infectious sporozoites, even if deployment is suboptimal. The consequences of modest reductions in hepatic stage parasite numbers might have disproportionate beneficial consequences on malaria morbidity, as suggested by the results of one of the Phase IIb trials of the anti-PE vaccine RTS,S [30,31]. Moreover, administration of this type of drug would not impede natural boosting of immune responses against PE or erythrocytic stage parasites.

In conclusion, the discovery of molecules with exclusive action against the hepatic phase of the life cycle lays the foundations to re-evaluate causal prophylaxis as a tool that can be incorporated in control strategies and enhance global efforts to reduce the major burden exacted by malaria.

## Supporting Information

Figure S1 Scheme of Fractionation from the Crude Methanolic Extract (35 KB PPT)

Figure S2 Scheme of Fractionation from the Decoction (25 KB PPT)

Table S1 Nuclear Magnetic Resonance Data for Tazopsine (36 KB PPT)

### Accession Numbers

The GeneBank (http://www.ncbi.nlm.nih.gov/Genbank) accession number for the *P. yoelii* 265BY 18S rRNA sequence is AF266261.

## Abbreviations

IC_50_: 50% inhibitory concentration
NCP-tazopsine: *N*-cyclopentyl-tazopsine
PE: pre-erythrocytic
PI: postinfection
TC_50_: 50% toxic concentration
TI: therapeutic index
